# Hydrogel treatment for idiopathic osteoarthritis in a Dunkin Hartley Guinea pig model

**DOI:** 10.1371/journal.pone.0278338

**Published:** 2022-11-30

**Authors:** Lauren R. Parola, Megan P. Pinette, Benedikt L. Proffen, Nicholas J. Sant, N. Padmini Karamchedu, Meggin Q. Costa, Janine Molino, Braden C. Fleming, Martha M. Murray

**Affiliations:** 1 Department of Orthopaedics, Warren Alpert Medical School of Brown University/ Rhode Island Hospital, Providence, RI, United States of America; 2 Department of Orthopaedic Surgery, Boston Children’s Hospital, Harvard Medical School, Boston, MA, United States of America; University of South Carolina, UNITED STATES

## Abstract

The study objective was to determine if intraarticular injections of an extracellular matrix (ECM) powder and blood composite (ECM-B) would have a significant impact on post-operative gait parameters without eliciting adverse cartilage changes or severe lymphatic reactions in an idiopathic osteoarthritis (OA) model. Twenty-one Dunkin Hartley Guinea pigs received an intraarticular injection of ECM-B in each knee and were split into sub-groups for gait assessment and post-harvest knee evaluations at 1 week (n = 5), 2 weeks (n = 5), 4 weeks (n = 5), or 8 weeks (n = 6). The results were compared with a control group (n = 5), which underwent bilateral injections of phosphate-buffered saline (PBS), gait measurements at 1, 2, 4, and 8 weeks, and post-mortem knee evaluation at 8 weeks post-injection. Hind limbs and popliteal lymph nodes were collected at the Week 8 endpoint and underwent histological analysis by a veterinary pathologist. Significant improvement in hind limb base of support was observed in the ECM-B group compared to the control group at Week 4 but was no longer significant by Week 8. No significant differences were observed between control and ECM-B groups in hind limb cartilage, synovium, or popliteal lymph node histology at Week 8. In conclusion, administration of an ECM-B material may improve gait for a limited time without significant adverse effects on the cartilage, synovium, or local lymph nodes.

## Introduction

Osteoarthritis (OA) is the most common form of arthritis in the United States [1]. Despite its frequency, there are few effective treatments for early-stage OA and most focus on alleviating symptoms. Corticosteroid injections or non-steroidal anti-inflammatory drugs (NSAIDs) have been used for pain management in osteoarthritis patients [2, 3]. While these treatments have been found to be effective in the short term to relieve pain, both have resulted in adverse effects including cartilage degradation when used long term [2, 3]. As an alternative, our lab has developed a new treatment for early-stage OA in the form of an injectable hydrogel made up of lyophilized extracellular matrix (ECM) proteins. At the time of treatment, the ECM solution is mixed with blood to form a collagen-fibrin hydrogel that, when injected into the knee, improved gait, and reduced macroscopic cartilage damage in a rat model of posttraumatic osteoarthritis [4]. Despite uncertainties regarding posttraumatic versus idiopathic OA progression, some evidence suggests similar components of pathogenesis [5]. Whether similar beneficial effects following the injectable hydrogel treatment would be seen in idiopathic osteoarthritis is unknown.

The aim of this study was to determine the efficacy of the ECM proteins and autologous blood (ECM-B) mixture in alleviating gait impairments without causing destruction of joint tissue in a spontaneous (idiopathic) OA model. The Dunkin Hartley (DH) Guinea pig was selected because of the histopathologic similarities between DH Guinea pig OA and human idiopathic OA [6]. Disease severity in DH Guinea pigs increases with age and therefore allows OA to be observed bilaterally without causing additional joint destruction by surgically inducing the disease [7, 8]. Microscopic OA-related changes to the cartilage and bone of DH Guinea pigs appear as early as three months of age and increase over time [7, 8]. The Guinea pigs at project commencement were six months old, the age at which this species reaches skeletal maturity [9]. Gait parameters were evaluated both before and after treatment [10]. We hypothesized that post-operative gait enhancements would be seen in DH Guinea pigs treated with the ECM-B composite relative to phosphate buffered saline (PBS) treated controls and that these improvements would not be time dependent. Additionally, we hypothesized that treatment with the ECM-B composite would not elicit microscopic cartilage changes or a reaction in the lymph nodes different from control animals. In addition, we wanted to document the presence of the ECM-B composite in the knee joint over time.

## Methods

### Regulatory approvals

Written approvals were acquired from the Institutional Animal Care and Use Committee (IACUC) of Rhode Island Hospital and the Animal Care and Use Review Office of the Department of Defense prior to beginning the study. The study was designed to meet the ARRIVE guidelines [11].

### Animal allocation and timeline

**Fig 1:**
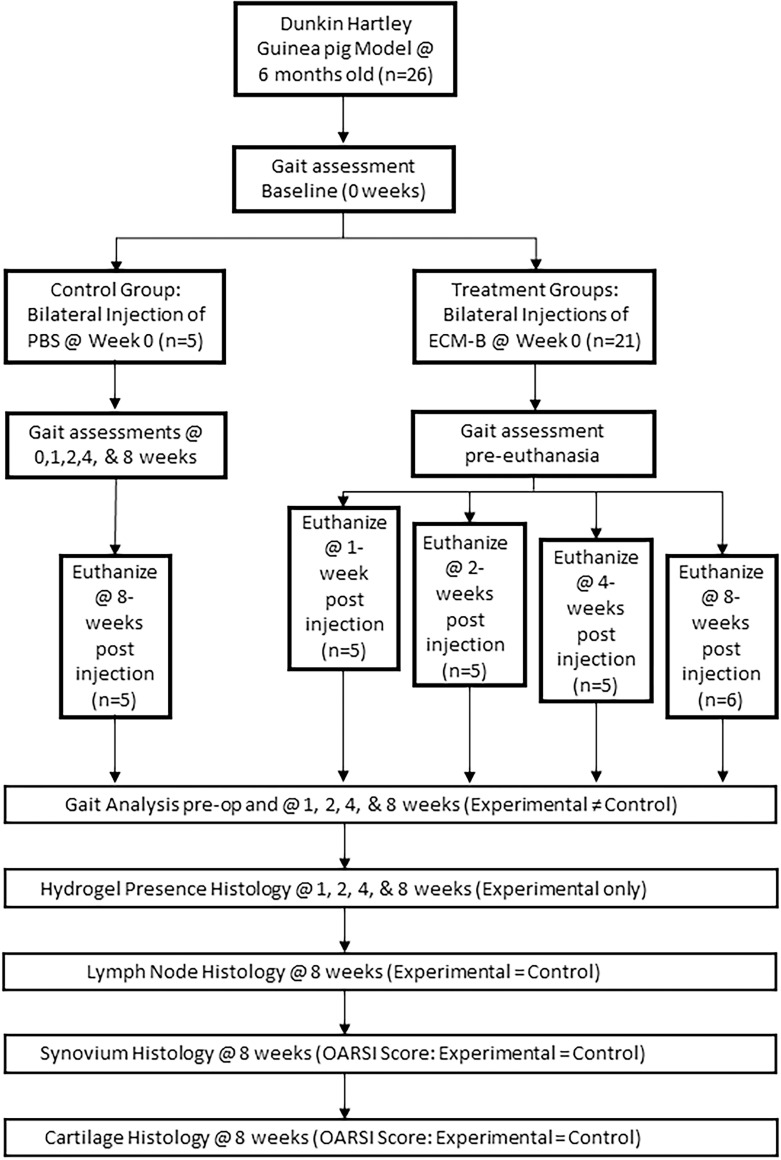
Schematic of experimental design.

Twenty-six male 6-month-old DH Guinea pigs (983.4±87.5 grams) were acquired (Charles River Laboratories, Wilmington, MA). The Guinea pigs were randomly divided into five groups (Fig 1) using dice. A sample size of at least 5 animals per group was utilized for this pilot study as it was deemed sufficient to look for large effects and to base future power calculations with this model. One group of five Guinea pigs was designated as the PBS-treated control group and the other 21 Guinea pigs received the ECM-B treatment. Within the ECM-B treatment group, animals were randomized to four timepoint groups. Depending on their timepoint assignment, the animals were euthanized at Week 1, Week 2, Week 4, and Week 8, respectively, while the control group animals were all euthanized at Week 8. Euthanasia was performed by an intravenous overdose of pentobarbital sodium solution (>120 mg/Kg) following anesthesia with isoflurane (1 to 4%, to effect). Control group animals underwent gait assessments at pre-injection, 1, 2, 4, and 8 weeks. ECM-B groups performed gait assessment at pre-injection and just prior to euthanasia. Gait parameters were studied as a primary outcome. The knee joints from animals euthanized at all time points underwent histological assessment to identify the presence of the ECM-B in the joint. Samples from Guinea pigs euthanized at 8 weeks were used to assess cartilage integrity and to study adverse reactions to the ECM-B composite in the synovium and lymph nodes as a secondary outcome. The 8-week animals were used for these comparisons as it was thought that the greatest changes would be evident at this time based on previous studies [12].

### Animal housing

All animals were housed at the Central Research Facility (CRF) at Rhode Island Hospital in groups of two or three animals. Animals were housed in modified rabbit cages filled with paper bedding (7099 TEK-Fresh Laboratory, ENVIGO). Cage bedding was changed 2–3 times per week. Guinea pigs were fed a standard diet (Certified Guinea Pig Diet Iradiated 50E6, Lab Diet, St. Louis, MO). Food and water were provided ad libitum. Housing facilities were kept on a light/dark cycle of 12 hours on/12 hours off daily. Guinea pigs were offered environmental enrichment in the form of food treats, bags of hay, and a large red plexiglass shelter placed in each cage. Animals were checked three times per week by lab staff, five times per week by veterinary staff, and daily by animal care facility staff.

### ECM-B powder production

The ECM powder was aseptically manufactured from reconstituted decellularized bovine connective tissue (Maverick BioSciences, New Zealand) by decellularizing, solubilizing and lyophilizing the tissue. The tissues were decellularized using Triton X-102 (Sigma-Aldrich, St Louis, MO), and then solubilized in an acidic pepsin solution to yield a protein content of >5mg/ml [13]. The slurry was brought to an alkaline pH using sodium hydroxide (Sigma-Aldrich) to inactivate pepsin and then neutralized with hydrochloric acid (Sigma Aldrich). The neutralized slurry was lyophilized, and the resulting dry ECM material was ground into a powder and loaded into 1 mL Luer lock syringes (125 mg per syringe). Each syringe was then placed into a double peel-pack package, which was stored at -20°C and protected from light until terminal sterilization. Terminal sterilization was performed by exposure to >20 kGy of electron beam irradiation at Steris, IL. ECM was prepared at Boston Children’s Hospital and transported to Rhode Island Hospital where it was mixed with the animal’s blood to form the ECM-B hydrogel at the time of intra-articular injection.

### Pre-procedure data collection

Prior to study commencement, the Guinea pigs were acclimated to the automated gait analysis walkway (Noldus CatWalk XT, Noldus Information Technology, Netherlands) on the system located in a CRF operating room [10]. The following week, baseline gait data for all 26 Guinea pigs were obtained over the course of two days. Prior to walking, each animal’s weight was recorded. Gait was recorded using the automated gait analysis walkway system to calculate the locomotion parameters (Table 1). Each animal’s paws were dampened using a water-soaked towel prior to the run so that the system would better identify the prints as the animal walked across the walkway. After the animal left the walkway, a lab member reviewed the data using the system’s “Auto Classify” function to identify each paw print in the trial. Incomplete prints and pauses were excluded from the run. Once classified, the number of step cycles in each run was recorded. Each Guinea pig walked until 15 complete step cycles (15 steps per each limb) had been acquired, which required multiple runs. The number of runs needed to reach 15 step cycles varied between animals, but an average of 4–5 runs was necessary.

**Table 1:** Parameters utilized to assess gait in statistical analyses. All four limbs were individually assessed (i.e., Right front limb, left hind limb).

Parameter	Description	Indication of Pain/OA	Source
**Stride Length (cm)**	Distance between successive prints for a given paw	Shorter stride length	[10, 16, 19]
**Swing Speed (cm/sec)**	Velocity of paw motion during the swing phase	Slower swing speed	[10, 16]
**Duty Cycle (%)**	Stand time as a percentage of step cycle	Smaller duty cycle	[16–19]
**Average Walking Speed (cm/sec)**	Distance an animal walked over time	Slower average walking speed	[10, 19]
**Hind Limb BOS (cm)**	Distance between the hind limbs during gait	Narrower hind limb BOS	[10]

### Treatment administration

At Week 0, all 26 animals underwent injections in both knees over the course of one day. Animals were taken from their housing, weighed, and put under anesthesia with isoflurane (1–4%, to effect) in a CRF operating room. Animals’ vitals were monitored and recorded every 15 minutes while under anesthesia. Their lower limbs were shaved and treated with betadine and 70% isopropyl alcohol. Each animal was then administered a dose of meloxicam (0.5 mg/Kg) via a subcutaneous injection for pain relief by a veterinary technician. One hundred twenty-five milligrams of the ECM powder were hydrated with 500 μl of sterile water and mixed back and forth to make 500 μl of a homogenous ECM suspension. One hundred microliter aliquots of ECM suspension were distributed into 1 mL Luer lock syringes. Two-hundred microliters of blood were drawn from each ECM-B animal’s jugular vein and immediately mixed with the 100 μl of ECM suspension to form a homogenous ECM-blood composite. After mixing the autologous blood with ECM, 200 μl of ECM-B was injected intra-articularly into both knee joints of the treatment group animals—100 μl in each individual knee joint. Control animals were administered 200 μl of sterile PBS into both knees. The order of treatment administration was randomized to prevent bias between groups. After injection, the animals were removed from isoflurane and placed in a recovery chamber until they were alert and then returned to the housing chamber. The animals were observed twice per day for three days after injection by the research team and the observations were documented.

### Post treatment data and sample collection

One week after treatment, the Week 1 ECM-B and control animals walked on the automated gait analysis walkway in a CRF operating room. Each animal was weighed, and their gait was recorded as was done at baseline. The next day, the Week 1 ECM-B animals were euthanized using isoflurane (1 to 4%, to effect) to first sedate each animal followed by a 3 mL intravenous overdose of pentobarbital sodium solution (>120 mg/Kg). The hind limbs and popliteal lymph nodes were harvested from each animal. The limbs were positioned at 70-degrees of flexion using a positioning block and fixed in 10% formalin solution in preparation for histology. After 24 hours, lymph nodes were transferred to 70% ethyl-alcohol solution. After 1 week, the hind limbs were also transferred to ethyl-alcohol. This process was repeated for the 2, 4, and 8-week animals. Eight weeks after initial treatment, both the control and the Week 8 ECM-B groups were humanely euthanized as described above, and their hind limbs and lymph nodes were harvested.

### Histology

After formalin fixation, both limb and lymph node samples were prepared for scoring. Knees were detached from the blocks, trimmed to fit in cassettes (Leica Biosystems, Buffalo Grove, Il), and sent in 70% alcohol to the COBRE Histology and Imaging Core at the University of New England. The limbs were decalcified in EDTA, embedded in paraffin, and 5 μm slices were collected every 200 μm in the coronal plane. Slicing continued through the joint until reaching the central section of the knee at the posterior edge of the anterior cruciate insertion on the tibia. Five slices were collected at this level. From the central section, one slide was stained with Toluidine blue for identifying proteoglycan content and another with Masson’s Trichrome to visualize collagen and the presence of ECM-B [6, 14, 15]. Lymph nodes were sent to the Lifespan Molecular Pathology Core for slicing and staining. Two slices were taken every 100 μm at 10 levels and one slice from each level was stained with hematoxylin and eosin stain.

Blinded stained slides from the central coronal section of the joint from all animals and the lymph node samples from Week 8 and control animals were then sent to an independent veterinary pathologist for scoring. Central coronal slice slides from the knee joints at all time points were assessed to identify ECM-B within the joint. Slides from control and Week 8 joints were further assessed using a semiquantitative OARSI scoring system recommended for Guinea pigs to identify microscopic cartilage changes and signs of synovitis reaction [6]. Stained lymph node slides from Week 8 ECM-B and control animals were scored by a veterinary pathologist using a semi-quantitative scoring system based on sinus drainage reactions.

### Gait analysis

Five parameters, which had been previously utilized to study quadruped gait, were selected to pinpoint abnormalities in the animal’s step cycle (Table 1) [10, 16–19]. These parameters include stride length (cm) [10, 16, 19], swing speed (cm/sec) [10, 16], duty cycle (%) [16–19], average walking speed (cm/sec) [10, 19], and hind limb base of support (BOS) (cm) [10].

### Statistical analysis

For the gait outcomes, all values were evaluated using mixed effects models (SAS Institute Inc. Cary, North Carolina). Classical sandwich estimators were used to protect against possible model misspecification. Pairwise comparisons between experimental conditions (i.e., group or time) were conducted via orthogonal contrasts. Adjustments for multiple comparisons were made using the Holm’s method to calculate adjusted P values (P adj). Means were considered significantly different if the adjusted P value was less than 0.05. After scoring, medial tibial plateau, inflammation, and lymph node data from control and Week 8 ECM-B animals were analyzed using generalized linear models to compare results between groups. The Holm test was used to calculate adjusted P values.

## Results

### General animal welfare

All Guinea pigs maintained good health throughout the study. Mean animal weight at the time of treatment was 983±87.5 grams. All but two animals gained weight over the course of the study. The two animals that lost weight only lost 3.5% or less of their body weight. No adverse health effects requiring early euthanasia were observed in any animal.

### Gait assessment

**Fig 2:**
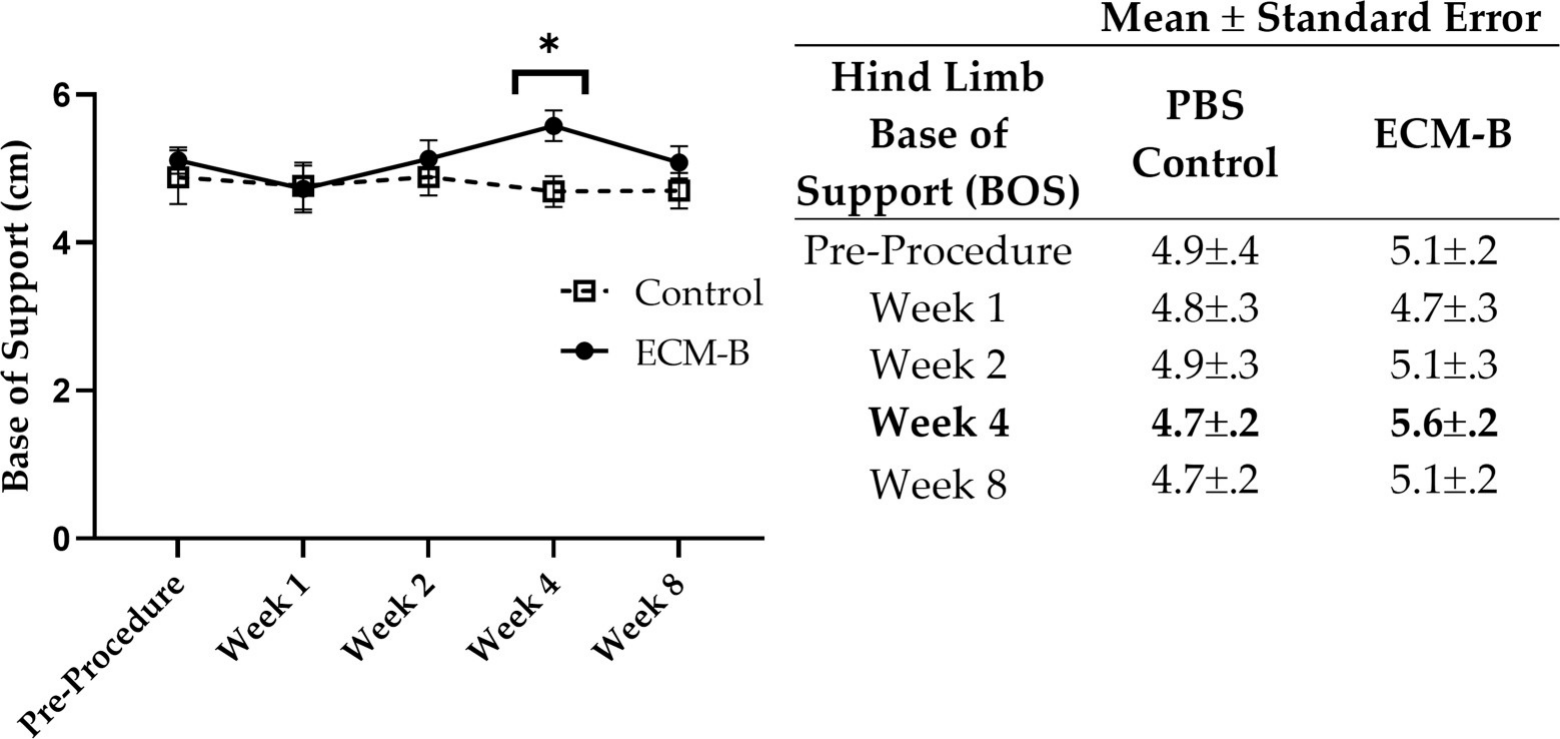
Hind limb BOS of ECM-B and control animals from pre-procedure to Week 8 post-injection. Circles represent means of the control group and squares represent ECM-B group means. The error bars represent the standard error. Mean and standard error values can be seen in the table to the right of the graph. Significantly different means are denoted by an asterisk in the graph and bolded in the table.

There was a significant effect of group on hind limb base of support (BOS). While there was no significant difference between groups at 1 and 2 weeks (P adj.>.05 for both comparisons), the hind limb BOS of the ECM-B animals was significantly wider (P adj = .0235) than that of the PBS-treated control animals by 4 weeks (Fig 2). There were no significant differences between the groups at 8 weeks after ECM-B injections (P adj. = .98). Therefore, the peak response in the ECM-B group occurred at 4 weeks. However, treatment group did not have a significant effect on swing speed, stride length, hind limb duty cycle, or average speed (Table 2, P adj.>.05).

**Table 2:** Mean gait parameter value of each group at each time point with standard error. Significant P values (P adj. < .05) are bolded. Each group contains n = 5 animals except for week 8 ECM-B where n = 6. All values are presented as the mean± standard error.

**Average Speed (cm/s)**	**Control**	**ECM-B**
**Pre-Procedure**	36.42±4.67	44.40±3.95
**Week 1**	40.72±3.95	37.05±3.95
**Week 2**	38.81±6.23	49.57±6.28
**Week 4**	44.12±3.84	36.92±3.84
**Week 8**	41.77±4.13	38.74±3.77
**Right Hind Swing Speed (cm/s)**	**Control**	**ECM-B**
**Pre-Procedure**	125.96±8.99	133.26±4.38
**Week 1**	131.49±7.05	116.94±7.05
**Week 2**	131.19±11.04	140.56±11.04
**Week 4**	135.27±7.77	118.7±7.77
**Week 8**	133.84±6.55	130.32±5.99
**Left Hind Swing Speed (cm/s)**	**Control**	**ECM-B**
**Pre-Procedure**	**110.81±8.83**	**131.5±3.33**
**Week 1**	122.49±8.21	115.10±8.21
**Week 2**	127.73±7.38	134.38±7.38
**Week 4**	134.80±6.22	114.4±6.22
**Week 8**	130.62±5.57	130.17±5.09
**Right Hind Stride Length (cm)**	**Control**	**ECM-B**
**Pre-Procedure**	16.73±1.00	17.79±0.49
**Week 1**	17.52±0.93	16.99±9l.93
**Week 2**	17.41±0.89	18.80±0.89
**Week 4**	17.72±0.56	16.63±0.56
**Week 8**	17.61±0.46	17.30±0.42
**Left Hind Stride Length (cm)**	**Control**	**ECM-B**
**Pre-Procedure**	16.79±0.98	17.94±0.48
**Week 1**	17.47±0.97	17.06±0.97
**Week 2**	16.99±1.00	18.69±1.00
**Week 4**	17.93±0.62	16.42+0.62
**Week 8**	17.48±0.51	17.26±0.47
**Right Hind Duty Cycle (%)**	**Control**	**ECM-B**
**Pre-Procedure**	68.16±3.26	63.64±1.59
**Week 1**	63.12±2.91	64.30±2.91
**Week 2**	65.18±2.90	60.72±2.90
**Week 4**	61.74±1.83	64.33±1.83
**Week 8**	64.20±1.93	66.38±1.76
**Left Hind Duty Cycle (%)**	**Control**	**ECM-B**
**Pre-Procedure**	63.27±4.13	62.24±2.01
**Week 1**	62.00±3.23	64.18±3.23
**Week 2**	64.38±4.46	59.54±4.46
**Week 4**	62.42±2.56	62.59±2.56
**Week 8**	63.52±2.29	66.39±2.09

### Presence of ECM-B material in the joint

**Fig 3:**
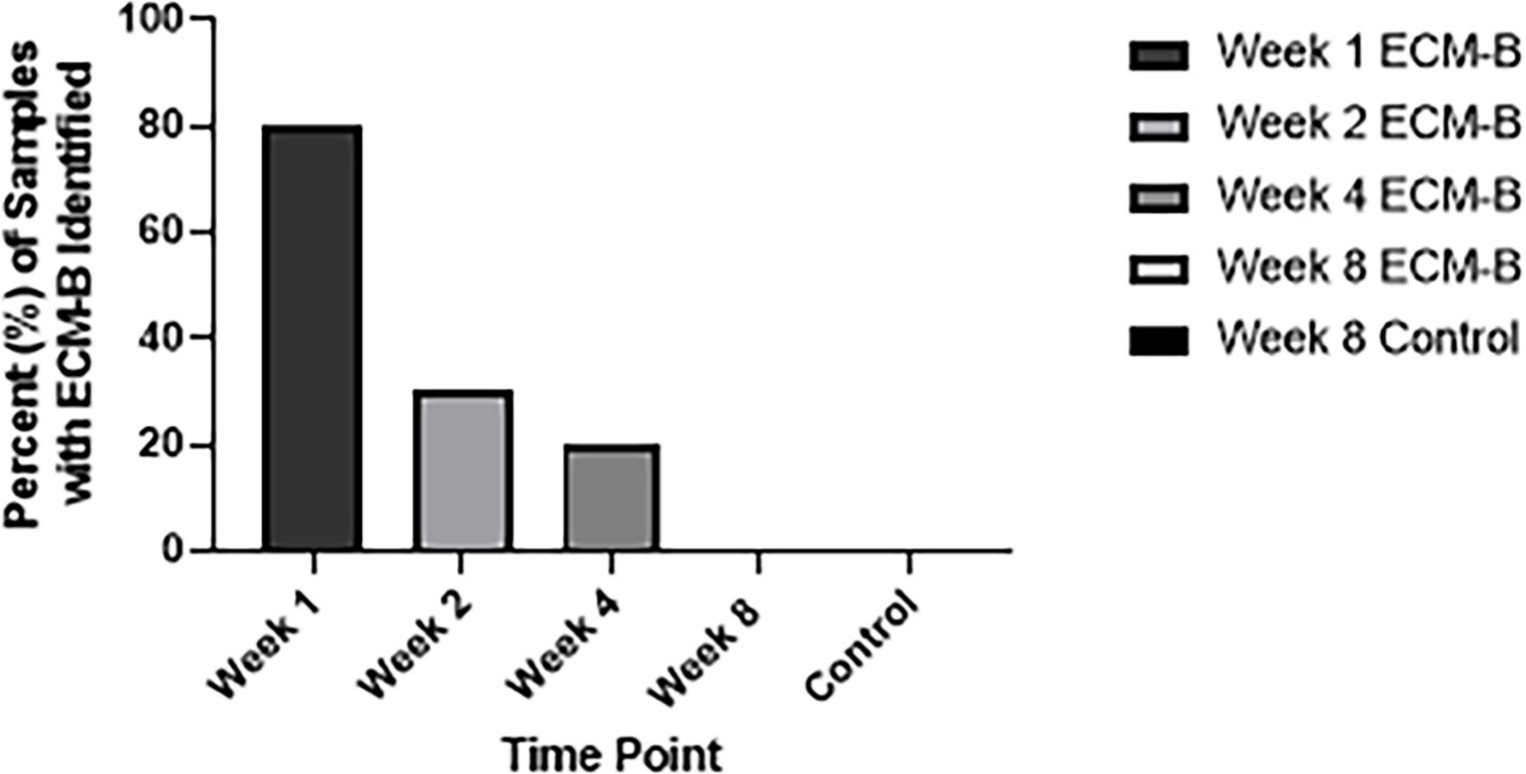
Percent of animals with ECM-B material present in the tibiofemoral joint at the time of harvest. Each ECM-B joint is seen in variable tones of gray and Week 8 control samples are black.

The proportion of animals with ECM-B material present in the tibiofemoral joint decreased over time. ECM-B material was noted in the joint space in 80% of Week 1 samples, 30% of Week 2 samples, 20% of Week 4 samples and 0% of Week 8 samples (Fig 3).

### Microscopic cartilage changes

Intra-articular ECM-B treatment resulted in microscopic cartilage scores that were not significantly different than PBS-treated control animals at 8 weeks. Significant differences were not observed in any of the parameters scored in the medial tibial plateau (MTP) including cartilage structure subscores, proteoglycan content subscores, cellularity subscores, tidemark integrity subscores, osteophyte subscores, and total scores (Table 3).

**Table 3:** Comparison of histology scores between ECM-B and PBS-treated control animals at eight weeks after treatment. Mean scores ± standard error are presented.

Variable	Control (n = 5)	ECM-B (n = 6)	Adjusted P-value
**MTP Articular Cartilage Score**	5.3±0.8	5.4±1.4	.81
**MTP Proteoglycan Content**	4.4±0.8	4.3±0.9	.85
**MTP Cellularity**	1.6±0.7	2.1±0.7	.10
**MTP Osteophyte Score**	0.7±0.7	1.3±0.6	.05
**Total MTP Score**	12.3±2.5	13.6±3.3	.28
**Chronic Inflammation**	0.9±0.6	1.3±0.6	.16
**MTP Tidemark Integrity**	0.3±0.5	0.5±0.5	.36
**Sinus Drainage Reaction**	0.8±0.8	1.0±0.9	.68

### Synovial reaction

There was no significant difference in inflammation scores for the synovium retrieved from the ECM-B group and PBS-treated control group joints at 8 weeks (Table 3).

### Lymph node reaction

Scoring of ECM-B and PBS-treated control group lymph nodes 8 weeks after treatment identified minimal to mild sinus drainage reactions that were not significantly different (Table 3).

## Discussion

The ECM-B treatment was presumed to be safe as no animals exhibited signs of pain or discomfort during observation in the days and weeks after treatment or lost an unhealthy amount of weight during the study. When comparing the average hind limb BOS between treatment groups at different time points, the BOS in ECM-B animals increased from Week 1 to Week 4 while the PBS-treated control animals’ BOS stayed constant. The hind limb BOS was significantly higher in ECM-B animals compared to control animals at Week 4. This wider BOS in ECM-B treated animals is consistent with prior work in OA-resistant Guinea pigs as well as in OA-prone Guinea pigs immediately after receiving an acute injection of anti-inflammatory medication to alleviate OA discomfort [10]. The work of Santangelo et al. established that a wider hind limb BOS is characteristic of a Guinea pig not experiencing OA-related discomfort [10]. In their work, there was an estimated difference of hind limb BOS means of 1.1 between OA resistant Guinea pigs and OA-prone Guinea pigs prior to treatment [10]. In this study, the estimated difference of hind limb BOS means between ECM-B treated animals and control animals was 0.89 at Week 4. Thus, the wider BOS observed in ECM-B animals could indicate that the treatment helped to alleviate symptoms of OA and allowed the animals to walk with a more normal gait.

During Week 8, BOS in the ECM-B animals was lower than that of the previous weeks and was not significantly different from the PBS-treated control group. The reduction in hind limb BOS at 8 weeks could indicate that the pain-alleviating effects of a single dose of ECM-B subsided by this time. This result is also supported by the work of Santangelo et. al., who found that the gait-enhancing effects of NSAID administration diminished by 72-hours after treatment as evidenced by a narrower hind limb base of support in DH Guinea pigs [10]. Although gait findings were not significant until Week 4, this result could indicate that the ECM-B could take a few weeks to reach its full pain relieving potential before reaching its therapeutic endpoint by Week 8, unlike the immediate effects of NSAID administration as seen in the previous work [10]. However, due to the limited size of this pilot study, it is important to consider that these small sample sizes may not have the power to detect these earlier effects.

When scoring a Masson’s Trichrome stained central osteochondral slice from each tibiofemoral joint, the veterinary pathologist, who was blinded to the treatment group, was unable to identify any ECM material from the Week 8 ECM-B treated animals. The ECM-B composite was, however, identified in 30% of Week 2 samples and 20% of Week 4 samples. With the positive gait enhancements seen in Week 4, despite less than half of the ECM-B composite being seen in the joint, this suggests that the entirety of the injectable does not need to be present for therapeutic benefits. This could establish a preliminary percentage threshold of material required for efficacy. Additionally, the lack of material in the joints during Week 8 adds further evidence that the treatment’s effect may have worn off by this time despite the initial positive gait enhancements seen at Week 4. Therefore, it may be beneficial to administer multiple ECM-B doses to maintain the positive therapeutic effects seen in the first 4 weeks after treatment. A longer-term investigation evaluating multiple doses of the ECM-B therapeutic has been planned. Furthermore, an increase in sample size may enable the detection of therapeutic effects at the earlier timepoints.

The ECM-B composite did not appear to have a detrimental impact on joint histology. After adjusting for pairwise comparisons, a significant difference in relevant scoring metrics was not identified between ECM-B and control samples at 8 weeks. The overall medial tibial compartment score of the ECM-B treated joints at 8 weeks after injection was not significantly different than that of the control animals. These results indicate that the treatment did not exacerbate the progression of OA in the joint. Additionally, no evidence of adverse reactions to the ECM-B treatment were seen in the synovium or lymph nodes. The chronic inflammation scores in ECM-B animals were not significantly different than that of the control animals, indicating ECM-B administration did not elicit a long-term inflammatory response. No significant difference was found in the sinus drainage reaction in popliteal lymph node scores from each treatment group, suggesting the ECM-B treatment did not cause an immune response different than that seen in the PBS-treated control animals. Overall, this study showed that the treatment did not elicit an abnormal reaction detrimental to the Guinea pigs’ health, modeling the safety of ECM-B for use in a synovial joint.

A significant difference was not found between groups for any other parameters that were analyzed (Table 2). However, there was a significant difference in the left hind limb swing speed pre-procedure. The reason for this is unclear as the animals were randomly assigned to treatment groups and underwent the same training protocol to familiarize them with the gait analysis system. We acknowledge multiple factors may have impacted the gait results. The cross-sectional design of this study resulted in inconsistencies in the walking schedule of each group and may have impacted gait results. Control animals walked at every time point while ECM-B animals only walked during pre-procedure (baseline) and their specified end point. Because control animals were better acquainted with the walkway, the resultant gait data were likely impacted. To avoid this problem in future work, all animals should be walked on the system at regular and consistent time points to maintain animal familiarity with the gait analysis walkway. We also acknowledge that age could have impacted the result. While previous work has reported that OA-related joint damage can be seen in DH Guinea pig as young as three months of age, more substantial cartilage fragmentation is present by 9 months of age [8]. Utilizing older animals in future studies may better demonstrate OA-related gait impairment and the positive impact of ECM-B treatment on articular cartilage integrity as seen previously in a rat ACL transection model [4]. Additionally, the results of this study were likely impacted by the small sample size. The sample size may have influenced the cartilage pathology results as a high degree of variability was observed in the pathology scoring of structural OA within groups. The sample size was selected to look for large differences and to serve as pilot data for future studies. Finally, when the study was designed, we aimed to detect more differences in gait and cartilage integrity due to ECM-B treatment as previously seen in a rat model of posttraumatic OA [4, 12]. It may be that ECM-B treatment has a greater effect for the early treatment of PTOA compared to idiopathic OA. Future studies need to be designed to further investigate this possibility.

This study was a pilot so there is a chance that the findings were incidental due to the small sample size as noted above. However, we have previously shown in the rat model that the ECM-B hydrogel slows the progression of posttraumatic osteoarthritis [4, 12]. Therefore, this pilot study was designed to evaluate the safety of the ECM-B and to see if the ECM-B has promise to provide relief to in a model of idiopathic osteoarthritis. The results of the study show that it is safe. The gait data from this idiopathic model suggest that there may be early pain-relieving effects (significant at 4 weeks but a trend at 1 and 4 weeks), but structural changes are not evident in the histology. Further work is needed. Given the more promising results in the previous posttraumatic osteoarthritis model, we are currently performing studies evaluating different dosing strategies to see if we can get a more robust effect on idiopathic osteoarthritis, and to define the mechanism of action.

Overall, this study showed promising preliminary results demonstrating the safety and potential efficacy of an ECM-B composite in the treatment of early-stage idiopathic osteoarthritis in DH Guinea pigs. While gait differences were unable to be identified between groups at every time point, a trend of increasing BOS over the first four weeks post-injection indicates that the treatment may help alleviate some OA-related gait impairment in DH Guinea pigs. In addition to relieving gait impairment, the intra-articular injections did not appear to have a detrimental impact on joint histology or overall animal health. While further studies are required to optimize the treatment regimen, these data indicate that ECM-B is deserving of further study as an alternative to corticosteroids for treatment of osteoarthritis pain without the detrimental impact on joint integrity seen with long-term corticosteroid usage [2, 3, 20].
